# Clinical predictors of dysphagia in patients with sepsis in a high-complexity hospital: insights for early identification

**DOI:** 10.1590/2317-1782/e20250112en

**Published:** 2026-01-26

**Authors:** Lorrayne Trapia de Paula, Fernanda Chiarion Sassi, Carina Escudero, Ana Paula Ritto, Juliana Helena Ferreira Zago Dib, Claudia Regina Furquim de Andrade

**Affiliations:** 1 Departamento de Fisioterapia, Fonoaudiologia e Terapia Ocupacional, Faculdade de Medicina, Universidade de São Paulo – USP - São Paulo (SP), Brasil.; 2 Divisão de Fonoaudiologia, Hospital das Clínicas, Faculdade de Medicina, Universidade de São Paulo – USP - São Paulo (SP), Brasil.; 3 Instituto Central, Hospital das Clínicas, Faculdade de Medicina, Universidade de São Paulo – USP - São Paulo (SP), Brasil.

**Keywords:** Deglutition Disorders, Deglutition, Swallowing, Sepsis, Critical Illness

## Abstract

**Purpose:**

To characterize the swallowing function of patients with sepsis, describe the frequency of oropharyngeal dysphagia, examine the rehabilitation duration required, and identify clinical predictors associated with the occurrence of dysphagia in a high-complexity hospital setting.

**Methods:**

This cross-sectional observational study included 35 patients diagnosed with sepsis, whose clinical severity was assessed using the National Early Warning Score (NEWS). Clinical and demographic data were collected from medical records. All participants underwent an initial screening with the Yale Swallow Protocol, and those identified at risk for dysphagia were further evaluated using the Dysphagia Risk Evaluation Protocol – Screening (DREP) and the Protocol for Introduction and Transition to Oral Feeding (PITOF). Swallowing functionality was classified using the Functional Oral Intake Scale (FOIS).

**Results:**

Dysphagic patients had pulmonary infection as the primary source of sepsis, higher clinical severity, a greater prevalence of comorbidities such as neurological diseases and heart conditions, lower FOIS scores, increased need for alternative feeding routes, longer hospital stays, and a higher risk of mortality. The most frequent clinical signs included prolonged oral transit time, reduced laryngeal elevation, and aspiration indicators such as throat clearing and coughing after swallowing.

**Conclusion:**

Oropharyngeal dysphagia in patients with sepsis is associated with worse clinical outcomes, emphasizing the importance of early diagnosis and specialized management.

## INTRODUCTION

Sepsis remains a major public health challenge and is one of the leading causes of intensive care unit (ICU) admissions, increased hospital costs, and high mortality rates^(1-3)^. It is characterized by organ and/or systemic dysfunction secondary to an abnormal immune response to infection, which, if not properly managed, can progress to septic shock, multiple organ failure, and death^(1,3,4)^. A study reported that the incidence of sepsis in ICUs can reach 36 cases per 1,000 patient-days, with a mortality rate of 55%^(2)^. In emergency departments, the prevalence is approximately 5.4 cases per 1,000 visits, with a mortality rate of 32%^(5)^.

Clinically, sepsis often presents with fever or hypothermia, tachycardia, tachypnea, hypotension, altered mental status, and multi-organ dysfunction^(4)^. The most common sources of infection include the lungs, urinary tract, bloodstream, and abdomen, with pneumonia accounting for approximately 48% of cases^(4,6)^.

Although swallowing impairments are well-documented in conditions such as neurological diseases, head and neck cancer, orotracheal intubation, and tracheostomy^(7)^, few studies have specifically addressed oropharyngeal dysphagia in patients with sepsis. A literature review identified one study^(8)^ that described epidemiological and clinical characteristics of critically ill patients and their oral feeding contraindications. Although 30% of the 128 patients evaluated were admitted due to sepsis, the study's heterogeneous sample did not allow for specific conclusions about dysphagia in this subgroup. Another study^(9)^ suggested a possible correlation between sepsis and oropharyngeal dysphagia, proposing that dysphagia may be an independent consequence of the significant muscle weakness observed in critically ill patients.

The complexity of sepsis, often accompanied by multiple comorbidities and organ dysfunction, highlights the need to investigate factors that affect patients’ functional status. A 2016 study^(10)^ found that clinical severity, particularly central nervous system impairment, is directly associated with swallowing dysfunction, as measured by the Sequential Organ Failure Assessment (SOFA) score. Additionally, the increasing global incidence of sepsis has drawn attention to its long-term sequelae, including physical, cognitive, and psychological complications, further emphasizing the importance of understanding swallowing impairments in this population. Early identification and management of dysphagia are critical to reducing reliance on alternative feeding routes and minimizing the risk of aspiration pneumonia—one of the most serious complications of oropharyngeal dysphagia^(8,11-15)^.

Swallowing assessment in critically ill patients is essential, given their elevated risk of aspiration due to altered consciousness, respiratory complications, and frequent need for orotracheal intubation and mechanical ventilation^(8,10,13,15)^. Speech-language pathology interventions have been shown to reduce hospital stays and prevent readmissions related to aspiration. Moreover, early dysphagia identification contributes to lowering healthcare costs, an increasingly relevant concern in overburdened health systems^(8,10-12,14)^.

Thus, this study aims to characterize the swallowing function of patients with sepsis, describe the frequency of oropharyngeal dysphagia, examine the rehabilitation duration required, and identify clinical predictors associated with the occurrence of dysphagia in a high-complexity hospital setting.

## METHODS

We conducted an observational study approved by the Research Ethics Committee of Hospital das Clínicas da Faculdade de Medicina da Universidade de São Paulo (CAPPesq Process no. 6,079,863). Data collection was based on a review of medical records from patients admitted to the Emergency Department of a high-complexity (tertiary/quaternary level) hospital with suspected or confirmed sepsis between January and September 2024. Data collection procedures began only after the Informed Consent was signed by the participants or their respective guardians.

### Participants

The participants were selected using a convenience sampling method, which included all consecutive patients who met the eligibility criteria during the data collection period. These patients were admitted to the Emergency Department of a high-complexity (tertiary/quaternary level) hospital with suspected or confirmed sepsis between January and September 2024.

Suspected sepsis refers to a clinical condition where a patient exhibits signs of infection, such as fever, increased heart rate, and respiratory rate, along with evidence of organ dysfunction. This diagnosis is typically based on clinical presentation and is often assessed using tools such as the National Early Warning Score (NEWS) scale^(16)^, which helps identify patients at risk of developing sepsis. On the other hand, confirmed sepsis is diagnosed when there is definitive laboratory evidence of infection, such as positive blood cultures, in addition to clinical signs of organ dysfunction. This distinction between suspected and confirmed sepsis is crucial, as confirmed sepsis is associated with a significantly higher risk of mortality and complications^(16)^.

The inclusion criteria for this study were: patients aged 18 years or older with suspected or confirmed sepsis, as identified by the NEWS scale; and a Glasgow Coma Scale score of 9 or higher at the time of swallowing evaluation by the speech-language pathologist. Exclusion criteria included: a history or current presence of tracheostomy; a diagnosis of esophageal dysphagia; and a history of surgical procedures, tumors, or structural abnormalities involving the head and neck region.

Although pre-existing oropharyngeal dysphagia could be considered a potential confounding factor, it was not used as an exclusion criterion. This decision was based on the practical difficulty of reliably identifying pre-existing dysphagia during hospitalization—particularly in acutely ill patients with incomplete medical records or impaired communication. Moreover, the aim of the study was to characterize swallowing function and identify clinical predictors of dysphagia in the context of sepsis, irrespective of whether the disorder was pre-existing or newly developed.

### Data collection

Data was collected from patient medical records to characterize the demographic and clinical profile of the participants. This included age, sex, previous comorbidities, body mass index (BMI), orotracheal intubation when necessary (including the duration of use), the time between sepsis diagnosis and swallowing evaluation, the number of speech-language pathology sessions for the introduction of a safe oral diet, the number of sessions for the removal of alternative feeding routes, and the outcome of the speech-language pathology intervention (discharge from speech-language therapy, hospital discharge, suspension of therapy due to worsening clinical condition, hospital transfer, or death).

### Sepsis

Sepsis diagnosis was made and documented in the medical records by the attending medical team, and its characterization included the collection of the National Early Warning Score (NEWS)^(16)^, along with information about antimicrobial therapy use and its duration. NEWS is a tool used for the early identification of clinical deterioration risk by evaluating six physiological parameters: respiratory rate, oxygen saturation, body temperature, systolic blood pressure, heart rate, and level of consciousness. The level of consciousness is assessed using the AVPU scale, which categorizes a patient's responsiveness into four levels:

- A (Alert): The patient is fully awake and aware of their surroundings;
- V (Verbal): The patient responds to verbal stimuli;
- P (Pain): The patient responds only to painful stimuli;
- U (Unresponsive): The patient does not respond to any stimuli.

Each of the six parameters in the NEWS score is assigned a score from 0 to 3, and the total score can range from 0 to 20. Higher scores indicate a greater risk of clinical deterioration and necessitate immediate intervention. In our institution, NEWS scores of ≥5 trigger the Sepsis Protocol and the transfer to the Clinical Emergency Room. In this study, the NEWS score was collected upon the patient's admission to the hospital.

### Clinical assessment of swallowing

All patients admitted to the Emergency Department with a presumptive or confirmed diagnosis of sepsis were screened for the risk of dysphagia by a speech-language pathologist. The first step of the screening involved a comprehensive assessment of the structures involved in the swallowing process, including a general examination, as well as an evaluation of respiration, speech, and voice, in addition to assessing the orofacial and cervical regions.

After completing the first step, patients who exhibited an appropriate level of alertness, preserved temporal and spatial orientation, facial symmetry, adequate mobility of the lips, tongue, and cheeks, and no signs of respiratory changes—such as respiratory rate outside baseline parameters—were subjected to the Water Glass Test, based on the Yale Swallow Protocol^(17)^.

The Water Glass Test involves the consumption of 90 mL of water without pauses, while the patient is monitored for signs of dysphagia, such as coughing, choking, or changes in voice during or after ingestion. If the patient shows signs of failure, they are considered at risk of aspiration and referred for a more detailed swallowing evaluation. The Yale Swallow Protocol^(17)^ is a standardized and more comprehensive version of the 3-Ounce Swallowing Test^(18)^, which includes cognitive screening and an evaluation of the oral mechanism to ensure that the patient is able to safely perform the test. Exclusion criteria include the inability to maintain alertness, pre-existing dysphagia, the presence of alternative feeding methods, medical restrictions on oral intake, restrictions on elevating the head of the bed to at least 30 degrees, and the presence of a tracheostomy. This protocol aims to improve the identification of aspiration risk, ensuring greater safety and accuracy in dysphagia screening.

Patients who passed the test were monitored for a period of 7 days to assess any potential clinical deterioration or development of swallowing-related complications. If no clinical worsening or swallowing complaints were observed during the monitoring period, they were discharged from speech therapy.

Patients who failed the Water Glass Test, experienced complications during the 7-day monitoring period, met at least one of the established risk criteria for dysphagia (i.e. voice quality, coughing, and choking)^(17)^, or were using alternative feeding methods, had a history of dysphagia, or neurological diseases, underwent a comprehensive speech therapy swallowing evaluation. This evaluation included the application of the Dysphagia Risk Evaluation Protocol – Screening (DREP)^(11,19)^ and the Protocol for Introduction and Transition to Oral Feeding (PITOF)^(20)^.

The DREP is indicated for early screening of dysphagia risk at the bedside^(11,19)^. Its application includes the administration of controlled volumes of water and puree^(4)^. The final result of the evaluation suggests whether the patient can receive larger volumes of liquids/foods and different food consistencies, as well as whether there is a need for monitoring to ensure safe feeding. The DREP has already been validated in the literature, demonstrating a sensitivity of 92.9%, specificity of 75.0%, positive predictive value (PPV) of 65.0%, negative predictive value (NPV) of 95.5%, and accuracy of 80.9%^(19)^.

The protocol is divided into two sections: a water swallowing test and a puree swallowing test, with results recorded as “pass” or “fail.” A study conducted in 2014^(21)^ investigated risk factors for dysphagia following prolonged orotracheal intubation based on findings from the DREP during a 5 mL water evaluation. The results showed that multiple swallows, altered cervical auscultation, changes in voice quality after swallowing, coughing, and choking were indicators of high dysphagia risk. In this study, the risk of aspiration pneumonia was considered when the patient exhibited changes in at least one of the following signs: voice quality, coughing, choking and altered cervical auscultation. The variable of multiple swallows was not included as a risk criterion, as it may represent a physiological adaptation to swallowing^(22)^. The next protocol used was the PITOF^(20)^, which is a protocol designed to complement the clinical evaluation of swallowing and manage dysphagia during the introduction and transition phases of oral feeding in the hospital setting. It is applied after the DREP, using foods and liquids of different consistencies and larger volumes. Its methodology incorporates signs and symptoms commonly observed in clinical practice, including the offering of a range of consistencies, and may involve the use of therapeutic techniques. For statistical analysis, the pass/fail criteria for each level evaluated were considered, including the use of therapeutic strategies.

At the end of the complete bedside swallowing evaluation, the functional level of oral intake was determined using the Functional Oral Intake Scale (FOIS). Developed by Crary et al.^(23)^, this scale is validated for assessing oral intake in patients with dysphagia following a stroke. It consists of seven levels, classifying the ability to swallow based on clinical aspects. The first three levels are related to non-oral feeding, while levels four through seven refer to varying degrees of oral intake. At the higher levels, both dietary adaptations and compensatory strategies used by patients are considered, always focusing on what the individual consumes daily by mouth. For this study, the Functional Oral FOIS level was determined after the Water Glass Test for patients who passed the screening. For those requiring a full swallowing evaluation, the FOIS level was assessed following the DREP and clinical swallowing assessment with PITOF, considering the same criteria established by the DREP.

### Data analysis

The collected data were subjected to statistical analysis using SPSS software version 29. Quantitative data were analyzed descriptively (mean, standard deviation, median, and interquartile range for continuous data; and total count and percentage for categorical data) and inferentially, comparing the results of dysphagic patients with those of patients exhibiting functional swallowing. The Mann-Whitney U test for independent samples was used for quantitative data, and Pearson’s Chi-square test was applied for categorical data. The significance level adopted for all analyses was 5%.

For statistical analysis, the functional levels of oral intake were grouped as follows: Level 1 (Levels 1 and 2) – no oral intake; Level 2 (Levels 3, 4, and 5) – some consistency by mouth; Level 3 (Levels 6 and 7) – functional swallowing. This clustering of Levels 3–5 was chosen because, despite their clinical heterogeneity, all three denote a transition to oral feeding—albeit with continued enteral supplementation—and thus share a common functional milestone. Moreover, combining these levels helped to mitigate sparse‐data issues in individual categories, preserving statistical power and ensuring more reliable comparisons. The FOIS classification was collected from the patient’s medical record at two distinct points: during the evaluation and swallowing rehabilitation outcome.

## RESULTS

In this study, 35 individuals admitted to the emergency department with a confirmed diagnosis of sepsis were included, with their demographic and clinical data presented in Table 1. Among them, 17 patients exhibited functional swallowing, while 18 were diagnosed with dysphagia. The most prevalent comorbidities in the overall group were renal diseases, diabetes mellitus, and smoking. A statistically significant difference was observed between the groups regarding the prevalence of underlying neurological diseases, which were more frequent among patients with dysphagia. Additionally, although the difference in age between the groups did not reach statistical significance (p = 0.057), patients with dysphagia tended to be older, suggesting a potential clinical relevance that warrants attention.

**Table 1:** Demographic and clinical data

Variables	Functional Swallowing (n=17)	Dysphagia (n=18)	Total (n=35)	*p-value*
Age (years)				
median	46.0 (39.0; 64.0)	59.0 (56.0; 69.0)	57.0 (43.0; 67.0)	0.057
Sex, n (%)				
Male	10 (58.8%)	6 (33.3%)	16 (45.7%)	0.130
Female	7 (41.2%)	12 (66.7%)	19 (54.3%)	
BMI				
median	24.0 (22.0; 26.7)	23.3 (19.5; 28.0)	23.9 (21.5; 28.0)	0.683
Previous Comorbidities, n (%)				
Cardiopathy	2 (11.8%)	6 (33.3%)	8 (22.9%)	0.129
Pulmonary Disease	2 (11.8%)	2 (11.1%)	4 (11.4%)	0.952
Kidney Disease	8 (47.1%)	9 (50.0%)	17 (48.6%)	0.862
Neurologic Disease	1 (5.9%)	8 (44.4%)	9 (25.7%)	0.009^*^
Rheumatic Disease	1 (5.9%)	2 (11.1%)	3 (8.6%)	0.581
Diabetes Mellitus	7 (41.2%)	8 (44.4%)	15 (42.9%)	0.845
Systemic Hypertension	7 (41.2%)	11 (61.1%)	18 (51.4%)	0.238
Obesity	-	1 (5.6%)	1 (2.9%)	0.324
Alcoholism	1 (5,9%)	1 (5.6%)	2 (5.7%)	0.967
Smoking	-	1 (5.6%)	1 (2.9%)	0.324
**ICU, n (%)**	6 (35.3%)	10 (55.6%)	16 (45.7%)	0.229
ICU stay (in days)				
median	6.0 (4.0; 8.0)	6.5 (5.0; 20.0)	6.5 (4.5; 11.0)	0.492
**OTI, n (%)**	-	9 (50.0%)	9 (25.7%)	<0.001*

* Significant difference between groups according to the Pearson Chi-square Test

U Test for independent samples

Caption: n = number of patients; % = percentage; BMI = body mass index; ICU = intensive care unit; OTI = orotracheal intubation

Table 2 presents the data related to sepsis. The most commonly scored parameters on the NEWS scale were heart rate, changes in systolic blood pressure, and alterations in oxygen saturation. Dysphagic patients showed higher scores in the "altered level of consciousness" parameter. The primary sources of sepsis identified were pulmonary and urinary tract infections, with a considerable prevalence in the dysphagic group. In this group, 50% of the patients had the lungs as the initial focus of sepsis when they presented to the emergency department.

**Table 2:** Characterization of Sepsis

Variable	Functional Swallowing (n=17)	Dysphagia (n=18)	Total (n=35)	*p-value*
Score NEWS				
median	7.0 (5.0; 8.0)	7.5 (6.0; 9.0)	7.0 (5.0; 9.0)	0.219

Parameters NEWS, n (%)				
Respiratory Frequency	13 (76.5%)	7 (38.9%)	20 (57.1%)	0.025*
Saturation O_2_	10 (58.8%)	13 (72.2%)	23 (65.7%)	0.404
Supplemental O_2_	2 (11.8%)	7 (38.9%)	9 (25.7%)	0.067
Temperature	4 (23.5%)	10 (55.6%)	14 (40.0%)	0.053
Systolic Blood Pressure	12 (70.6%)	11 (61.1%)	23 (65.7%)	0.555
Heart Rate	14 (82.4%)	11 (61.1%)	25 (71.4%)	0.164
Level of Consciousness	2 (11.8%)	8 (44.4%)	10 (28.6%)	0.032^*^

Source of Sepsis, n (%)				
Abdominal	-	1 (5.6%)	1 (2.9%)	0.029*
Bloodstream	4 (23.5%)	2 (11.1%)	6 (17.1%)	
Skin/Soft tissue	5 (29.4%)	-	5 (14.3%)	
Bloodstream + Pulmonary	-	1 (5.6%%)	1 (2.9%)	
Pulmonary	2 (11.8%)	9 (50.0%)	11 (31.4%)	
Pulmonary + Urinary Tract	-	2 (11.1%)	2 (5.7%)	
Central Nervous System	1 (5.9%)	-	1 (2.9%)	
Urinary Tract	5 (29.4%)	3 (16.7%)	8 (22.9%)	

Duration of antimicrobial use, in days				
median	7.0 (5.0; 17.0)	9.5 (5.0;15.0)	9.0 (5.0; 17.0)	0.568
**Septic Shock, n (%)**	3 (17.6%)	7 (38.9%)	10 (28.6%)	0.164

* Significant difference between groups according to the Pearson Chi-square Test

Caption: n = number of patients; % = percentage; O_2_ = oxygen

Table 3 presents the swallowing classification of the groups according to the FOIS scale. In the dysphagia group, all 18 individuals were identified as at risk for dysphagia using the DREP protocol and underwent swallowing evaluation. Most patients in this group required an alternative feeding method (AFM), with a median of four interventions before discontinuation of this route. In contrast, no individuals in the functional group required AFM, which prevented direct comparisons between the groups.

**Table 3:** Functional Oral Intake Scale (FOIS)

Level	Functional Swallowing n = 17(%)	Dysphagia n = 18 (%)	Total n = 35 (%)	*p-value*
Initial Assessment				
1	-	1 (5.6%)	1 (2.9%)	<0.001^*^
2	-	6 (33.3%)	6 (17.1%)	
3	-	4 (22.2%)	4 (11.4%)	
4	-	2 (11.1%)	2 (5.7%)	
5	-	4 (22.2%)	2 (5.7%)	
6	1 (5.9%)	1 (5.6%)	2 (5.7%)	
7	16 (94.1%)	-	16 (45.7%)	

Outcome				
1	-	2 (11.1%)	2 (5.7%)	0.001*
2	-	2 (11.1%)	2 (5.7%)	
3	-	3 (16.7%)	3 (8.6%)	
4	-	1 (5.6%)	1 (2.9%)	
5	-	2 (11.1%)	2 (5.7%)	
6	1 (5.9%)	5 (27.8%)	6 (17.1%)	
7	16 (94.1%)	3 (16.7%)	19 (54.3%)	

* Significant difference between groups according to the Pearson Chi-square Test

Caption: n = number of patients; % = percentage

Table 4 presents the clinical outcomes and swallowing rehabilitation variables. All patients with functional swallowing met the criteria for discharge from rehabilitation; however, following the established protocol, they were monitored for seven days. Of these, 52.9% remained clinically stable and were discharged from the hospital before this period, while the remaining patients were discharged from swallowing rehabilitation after the full seven days. An association was identified between the presence of dysphagia and mortality.

**Table 4:** Clinical outcome and swallowing rehabilitation variables

Variable	Functional Swallowing (n=17)	Dysphagia (n=18)	Total (n=35)	*p-value*
Clinical outcome, n (%)				
Swallowing rehabilitation discharge	8 (47.1%)	7 (38.9%)	15 (42.9%)	0.030^*^
Hospital discharge	9 (52.9%)	4 (22.2%)	13 (37.1%)	
Hospital transfer	-	1 (5.6%)	1 (2.9%)	
Death	-	6 (33.3%)	6 (17.1%)	

Swallowing rehabilitation, in days				
median	5.0 (2.0; 7.0)	8.0 (4.0; 22.0)	7.0 (2.0; 8.0)	0.014^**^

Time between diagnosis of sepsis and swallowing assessment, in days				
median	4.0 (1.0; 5.0)	5.0 (1.0; 8.0)	4.0 (1.0; 7.0)	0.335
**Use of AFM, n (%)**	-	13 (72.2%)	13 (37.1%)	

* Significant difference between groups according to the Pearson Chi-square Test

** Significant difference between groups according to the Mann-Whitney U Test for independent samples

Caption: n = number of patients; % = percentage; AFM = alternative feeding method

## DISCUSSION

This study investigated the relationship between sepsis and oropharyngeal dysphagia, highlighting significant differences between the functional and dysphagic groups. The dysphagic group presented with greater clinical severity, evidenced by higher median scores on the NEWS scale, a higher frequency of pulmonary and urinary tract infections, and longer speech-language therapy follow-up. These findings are consistent with previous studies, such as Zielske et al.^(24)^, which emphasized the association between pulmonary infections and an increased risk of dysphagia in sepsis patients. The prolonged follow-up in the dysphagic group suggests the need for sustained intervention to address both the underlying infection and its impact on swallowing function, emphasizing the importance of interdisciplinary care, particularly speech-language pathologists, in optimizing recovery and preventing further complications.

Sepsis screening has evolved over the years, transitioning from the low-specificity Systemic Inflammatory Response Syndrome (SIRS) criteria to the Sequential Organ Failure Assessment (SOFA) and quick SOFA (qSOFA) scores, introduced by the Sepsis-3 guidelines in 2016^(1)^. However, qSOFA has demonstrated low sensitivity, prompting the Sepsis Surviving Campaign^(3)^ to recommend its use in combination with SIRS. Additionally, the National Early Warning Score (NEWS)^(16)^ has proven effective in the early identification of sepsis, exhibiting sensitivity comparable to SIRS and specificity similar to qSOFA. Therefore, an integrated approach based on multiple criteria is essential for achieving a more accurate diagnosis^(1,3)^.

Patients in the dysphagic group demonstrated greater heterogeneity in their levels on the FOIS scale, with a predominance of dietary restrictions and dependence on alternative feeding methods. A substantial proportion of patients with dysphagia was observed, along with a longer duration of speech-language pathology follow-up in this group. These findings may be associated with the clinical complexity of the affected individuals, particularly considering the systemic impact of sepsis on swallowing function. The extended duration of follow-up may reflect the need for more extensive and individualized therapeutic interventions and underscores the importance of early dysphagia assessment and management in sepsis patients to improve clinical outcomes and reduce complications^(25)^.

Sepsis, one of the leading causes of ICU admission, significantly affects swallowing function, particularly in patients requiring mechanical ventilation. Prolonged ventilation contributes to muscle weakness, which compromises swallowing function and increases the risk of aspiration and pneumonia^(14,21,26,27)^. This highlights the need for early and specialized interventions to minimize the impacts of dysphagia in critically ill patients. In this context, speech-language pathology plays a crucial role in managing dysphagia and supporting functional rehabilitation.

Our sample revealed that the most common comorbidities were diabetes mellitus, hypertension, and chronic kidney disease (CKD), with similar prevalence in both groups. However, the dysphagic group had a higher median age and a greater incidence of neurological and heart conditions, which are often linked to poorer outcomes in critically ill patients^(28)^. Some studies indicate that CKD is a significant risk factor for healthcare-associated infections, including UTIs and pneumonia, which were more prevalent in our dysphagic group^(29,30)^. The increasing prevalence of these chronic diseases, combined with population aging, contributes to greater vulnerability among older adults and exacerbates the severity of sepsis and its complications. The interaction between CKD, hypertension, and diabetes increases vulnerability to infections and exacerbates the severity of sepsis and its complications. These findings suggest that the presence of such comorbidities should be considered an important clinical warning in the early assessment of dysphagia risk in patients with sepsis. Rigorous clinical surveillance and early management of these conditions are essential to mitigate complications and improve outcomes in critically ill patients, reinforcing the need for targeted monitoring and timely intervention to optimize clinical trajectories.

Although few studies specifically address the relationship between sepsis and dysphagia, existing literature shows variation in the prevalence of oropharyngeal dysphagia within this population^(24,27,28)^. This variation is influenced by differences in assessment methods, with some studies emphasizing the need for standardized diagnostic and management protocols^(25)^. In our study, the NEWS scale was used to define sepsis and stratify the clinical risk of patients, with the dysphagic group showing a slightly higher median score, indicating greater clinical severity and the need for more intensive interventions. Infection sites differed between the groups, with pulmonary infections predominating in the dysphagic group, possibly linked to a higher incidence of respiratory complications, such as aspiration.

An important question raised by this study is the direction of causality between sepsis and dysphagia. Literature suggests that muscle weakness from prolonged mechanical ventilation may be a key factor in the development of dysphagia, but it remains unclear whether sepsis itself directly contributes to dysphagia. Could pulmonary sepsis predispose patients to dysphagia due to respiratory instability and prolonged mechanical ventilation? Or is dysphagia a contributor to sepsis through complications like aspiration pneumonia? Both processes may interact bidirectionally, exacerbating each other’s complications.

Pre-existing comorbidities, such as neurological diseases and heart conditions, significantly influence the interaction between sepsis and dysphagia. Previous studies highlight that muscle weakness and clinical instability are critical factors contributing to dysphagia in critically ill patients^(27,28)^. In our sample, swallowing data revealed significant differences between the groups. All individuals in the functional group exhibited functional swallowing, while the dysphagic group showed greater heterogeneity in FOIS scale levels, with a substantial proportion requiring alternative feeding methods. This clinical complexity necessitates dietary adaptations and prolonged nutritional support, emphasizing the need for further research to explore the interactions between sepsis, dysphagia, and associated complications.

The most frequent clinical findings in the dysphagic group included prolonged oral transit time, altered laryngeal elevation, and signs of aspiration, such as throat clearing and coughing after swallowing. These signs are important predictors of dysphagia and an increased risk of respiratory complications, including aspiration pneumonia^(21)^. However, the presence of these signs may vary depending on the severity of the underlying condition, such as sepsis or other associated comorbidities.

Finally, clinical outcomes revealed a higher mortality rate in the dysphagic group, while the functional group showed a higher frequency of early hospital discharge. Dysphagia appears to be associated with a higher risk of mortality, reinforcing the need for early identification and management to improve clinical outcomes and reduce complications. Although the age difference between groups did not reach statistical significance, the dysphagic group tended to be older (p = 0.057), suggesting that age may be a clinically relevant factor in the onset or worsening of swallowing dysfunction in sepsis. Advancing age is associated with physiological changes in swallowing, reduced muscle reserve, and a higher prevalence of chronic diseases, all of which may increase the vulnerability of older adults to complications such as dysphagia, aspiration, and poorer recovery outcomes^(7,8,10)^. Thus, even borderline differences in age should prompt clinical attention and proactive screening in older septic patients.

This study presents some limitations that should be considered. The relatively small sample size (n = 35) may affect the generalizability of the findings; however, the inclusion of a well-defined cohort of septic patients in a high-complexity hospital enhances the clinical relevance of the results. Data collection relied on medical records, which may introduce information bias; nonetheless, standardized clinical protocols were used for swallowing assessment, reducing variability. The absence of instrumental swallowing assessments, such as videofluoroscopy or fiberoptic endoscopic evaluation of swallowing (FEES), limits the direct confirmation of aspiration events. However, the study applied validated clinical screening and assessment tools to ensure a systematic evaluation of dysphagia risk. Additionally, while potential confounders such as disease severity, comorbidities, and nutritional support were analyzed, other factors—including variations in clinical management, medication effects, and respiratory support—may have influenced the outcomes. Future studies should employ larger cohorts and integrate instrumental swallowing assessments to refine early identification strategies and optimize dysphagia management in septic patients. Despite these limitations, the present study contributes valuable insights into the relationship between sepsis and swallowing dysfunction, reinforcing the need for systematic screening and targeted interventions in this high-risk population.

## CONCLUSION

In conclusion, this study, conducted with a convenience sample, demonstrates that oropharyngeal dysphagia in patients with sepsis is significantly associated with several risk factors, including comorbidities such as diabetes mellitus, hypertension, chronic kidney disease, and neurological disorders, as well as pulmonary and urinary tract infections. Patients with dysphagia exhibited characteristic impairments in swallowing function, such as dietary restrictions, dependence on alternative feeding methods, and clinical signs including prolonged oral transit time and altered laryngeal elevation. They also showed higher scores on the NEWS scale, prolonged hospital stays, and an increased risk of mortality. Notably, age emerged as a relevant factor, with older patients being more frequently affected by dysphagia, likely due to their increased vulnerability to infections and multimorbidity. These findings highlight the importance of early identification and targeted management of dysphagia to prevent complications, reduce hospitalization time, and improve survival in this high-risk population.
